# Arginine metabolism and neurocognitive impairment in offspring of bipolar parents: a high-risk case-control study

**DOI:** 10.3389/fpsyt.2025.1511397

**Published:** 2025-02-17

**Authors:** Gökçeçiçek Arıcı Sağlıyan, Fatih Hilmi Çetin, Fikret Akyürek, Oğuzhan Tok, Özlem Çiçek Zekey, Mustafa Esad Tezcan, Bilal Sağlıyan, Serhat Türkoğlu, Halit Necmi Uçar, Bahadır Öztürk, Kürşat Altınbaş

**Affiliations:** ^1^ Department of Child and Adolescent Psychiatry, Konya City Hospital, Konya, Türkiye; ^2^ Department of Child and Adolescent Psychiatry, Diamind Mental Academy, Konya, Türkiye; ^3^ Department of Medical Biochemistry, Selcuk University Faculty of Medicine, Konya, Türkiye; ^4^ Department of Child and Adolescent Psychiatry, Sivas Numune Hospital, Sivas, Türkiye; ^5^ Department of Child and Adolescent Psychiatry, Selcuk University Faculty of Medicine, Konya, Türkiye; ^6^ Department of Psychiatry, Faculty of Medicine, Selcuk University, Konya, Türkiye; ^7^ Private Practitioner, Konya, Türkiye

**Keywords:** arginine, asymmetric dimethylarginine (ADMA), bipolar disorder, children, neurocognitive function, high risk, offspring

## Abstract

**Introduction:**

The aim of this study is to investigate whether arginine and its metabolites can be an endophenotype for bipolar disorder (BD) and to evaluate the role of arginine metabolites and neurocognitive function levels in unaffected healthy children of parents diagnosed with BD in cognitive impairment.

**Methods:**

The study included 37 healthy children of parents diagnosed with BD Type I as the high-risk group and 36 healthy children of parents without any psychiatric disorders as the control group. The arginine, n-monomethyl-l-arginine acetate (L-NMMA), asymmetric dimethylarginine (ADMA), symmetric dimethylarginine (SDMA), citrulline, homoarginine, ornithine serum levels, and nitric oxide synthase (NOS) activity level of both groups were compared.

**Results:**

The study found that in the high-risk group, ADMA, SDMA, and ornithine levels were significantly higher compared to the controls, while citrulline and NOS activity level were significantly lower in the high-risk group compared to the controls. All neurocognitive performances of the high-risk group were considered statistically significantly worse compared to controls. The impairment in neurocognitive functions in the high-risk group was found to be correlated with ADMA, L-NMMA, citrulline, homoarginine, ornithine levels, and NOS activity level.

**Discussion:**

These findings highlight a potential link between arginine metabolism and executive dysfunction in individuals at high risk for BD. Further longitudinal studies are essential to fully understand the complex interactions between these factors.

## Introduction

Bipolar disorder (BD) is a chronic and severe psychiatric condition characterized by recurrent episodes of mania, hypomania, and depression, which significantly impact daily functioning. BD is known to have a strong genetic component, with heritability estimates suggesting that up to 85% of the variability in risk is attributable to genetic factors (1, 2). Beyond mood disturbances, individuals with BD often exhibit various neurocognitive deficits, including impairments in executive function, working memory, and attention. Notably, such cognitive deficits are also observed in the unaffected first-degree relatives of BD patients, which suggests these impairments may serve as potential endophenotypes that reflect underlying genetic vulnerability to the disorder (3).

**Figure 1:**
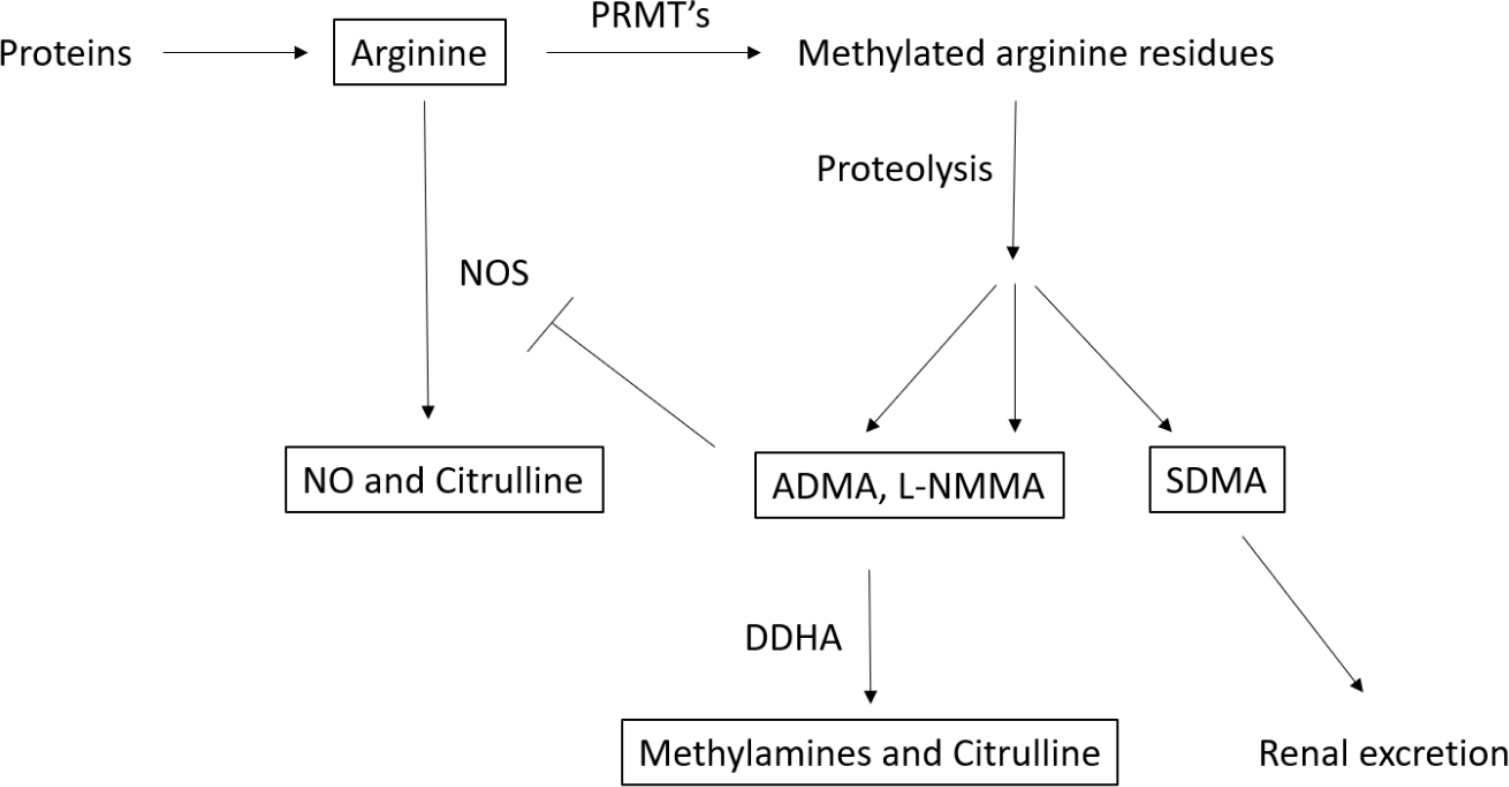
Metabolism of arginine and metabolites (18). PRMT’s, protein-arginine methyl transferase L-NMMA, n-monomethyl-l-argınıne acetate; ADMA, Asymmetric dimethylarginine; SDMA, Symmetric Dimethylarginine; NO, Nitric Oxide.

The etiology of BD is multifactorial, involving a complex interaction of genetic, biochemical, environmental, and psychosocial factors. Recent research has highlighted the potential role of arginine and its metabolites in the pathophysiology of BD, particularly in relation to neurocognitive impairments (4). Elevated levels of methylarginines, especially asymmetric dimethylarginine (ADMA), have been observed in individuals with BD, and higher concentrations of ADMA have been linked to poorer performance on cognitive tasks involving executive function and attention (5). These findings suggest that disruptions in arginine metabolism may contribute to the cognitive deficits associated with BD. Available clinical evidence has demonstrated that the nitric oxide (NO) signaling pathway is associated with schizophrenia, anxiety disorders, and affective disorders (6). NO a key signaling molecule synthesized from arginine, has garnered significant interest in the context of BD. NO is an important messenger in brain signaling and influences the balance ofmonoaminergic and glutamatergic neurotransmission. NO is involved in various neural processes, including synaptic plasticity, neuroinflammation, neurotransmission, and the regulation of learning and memory. Disruptions in NO production and associated pathways have been implicated in the pathophysiology of BD (7). A meta-analysis has demonstrated that NO levels change in individuals with BD (8). Arginine is a semi-essential amino acid and a substrate of important metabolic pathways in the physiological processes of the central nervous system and immune defense (9, 10). Arginine is transformed into NO and citrulline by endothelial nitric oxide synthase (eNOS), inducible nitric oxide synthase (iNOS) and neuronal nitric oxide synthase (nNOS) (11). Arginine, the precursor molecule for NO synthesis, plays a crucial role in maintaining NO levels in the brain. In BD, abnormalities in arginine metabolism may lead to altered NO signaling, contributing to both mood dysregulation and cognitive deficits. Additionally, interactions between the arginine-vasopressin pathway and NO synthesis are thought to influence mood regulation and symptom expression in BD. Arginine plays a crucial role in neuroinflammatory processes, particularly through its involvement in microglial activation. During this process, arginine serves as a substrate for the production of NO and reactive nitrogen species (RNS). The dysregulation of NO production significantly influences the progression of neuroinflammation. While excessive NO levels induce neurotoxicity by triggering the release of pro-inflammatory cytokines such as interleukin-6, tumor necrosis factor-alpha, and interleukin-1 beta, insufficient NO production can also have profound consequences. These cytokines disrupt synaptic function, exacerbate glutamate-mediated excitotoxicity, and increase blood-brain barrier permeability, thereby facilitating the infiltration of peripheral immune cells into the central nervous system and amplifying inflammation (12, 13). Conversely, low NO levels also play a pivotal role in neuroinflammation. Under physiological conditions, NO is essential for neuronal protection and the regulation of immune responses. However, reduced NO production can lead to an imbalance in microglial activity and impair resolution mechanisms necessary for controlling inflammation. The deficiency of NO has been associated with an increased accumulation of reactive oxygen species, thereby exacerbating oxidative stress and contributing to the persistence of neuroinflammatory processes (14, 15). Arginine metabolism generates key biomarkers that provide insights into these mechanisms. NO synthesis via iNOS has been shown to exert both protective and detrimental effects in the context of neuroinflammation. Notably, insufficient NO levels may impair anti-inflammatory responses and weaken cellular defense mechanisms. Arginase competes with NOS for arginine, converting it into ornithine and polyamines. Elevated arginase activity reduces NO production while promoting polyamine accumulation, a process linked to oxidative stress and inflammatory responses (16). Methylarginines, including ADMA and symmetric dimethylarginine (SDMA), are formed through the degradation of proteins containing arginine residues (see **Figure 1**). These molecules play important roles in regulating NO synthesis and cellular signaling pathways. ADMA, in particular, inhibits nitric oxide synthase, thereby reducing NO production. Elevated levels of ADMA have been associated with increased oxidative stress and neuroinflammation, which are believed to contribute to cognitive impairment in BD. Although the precise mechanisms through which methylarginines influence neurocognitive function remain unclear, their role in modulating NO levels, oxidative stress, and inflammation suggests they may be key contributors to the cognitive deficits seen in BD (17).

Patients with bipolar disorder frequently show cognitive dysfunction even during euthymia (3, 19). Areas of cognitive dysfunction in bipolar disorder include attention, language learning, memory and executive function (20, 21). Neurocognitive impairments have also been observed in the offspring of individuals with BD. Studies have demonstrated that children of BD patients tend to have lower intelligence quotients (IQ) compared to controls (22). Additionally, these children exhibit poorer cognitive performance on tasks involving processing speed and visual memory (23). It has been proposed that these cognitive deficits in the healthy offspring of BD patients may be related to disruptions in arginine metabolism, though this hypothesis has yet to be fully explored.

To date, no studies have specifically examined the relationship between arginine metabolites and executive function in the healthy offspring of BD patients. Given the role of arginine and its metabolites in NO regulation, oxidative stress, and neuroinflammation, it is plausible that abnormal arginine metabolism could contribute to the cognitive impairments observed in this at-risk population. Therefore, the present study aims to investigate the association between arginine metabolite levels and executive function in the healthy offspring of individuals with BD. Furthermore, we seek to determine whether elevated levels of methylarginines, such as ADMA and SDMA, are linked to cognitive impairments, and whether these molecules may serve as potential endophenotypes for BD. By elucidating the biochemical underpinnings of cognitive deficits in this population, this study may provide valuable insights into the biological factors that predispose individuals to BD and its associated cognitive challenges.

## Method

This study was conducted in collaboration with the Child and Adolescent Psychiatry Department, the Psychiatry Department, and the Medical Biochemistry Department at Selçuk University Faculty of Medicine Hospital. Consent was obtained from both the participating children/adolescents and their parents to voluntarily partake in the study. The sample for the study included 37 children and adolescents, aged 8-17, who had a parent diagnosed with bipolar I disorder according to DSM-5 (The Diagnostic and Statistical Manual of Mental Disorders) criteria following psychiatric interviews at the Psychiatry Clinic between June 2022 and December 2022, and who met the inclusion and exclusion criteria.

The control group consisted of 36 healthy volunteers, aged 8-17, who attended the Child and Adolescent Psychiatry Clinic at Selçuk University Faculty of Medicine Hospital for psychiatric consultation, and who did not have any psychiatric or other medical disorders. These controls were matched with the high-risk group based on age and sex and participated on a voluntary basis.

For all participants, medical history was taken to assess whether they met the inclusion and exclusion criteria. The Sociodemographic Data Questionnaire and Schedule for Affective Disorders and Schizophrenia for School Age Children-Present and Lifetime Version-Turkish Version (K-SADS-PL-DSM-5-T), stroop test, serial digit learning test (SDLT), and cancellation test (CT) were applied to the high-risk and control groups. Serum levels of arginine, N-monomethyl-L-arginine acetate (L-NMMA), ADMA, SDMA, citrulline, homoarginine, and ornithine, as well as the citrulline/arginine ratio reflecting NOS activity, were compared between the high-risk and control groups.

The high-risk group consisted of children aged 8-17 who had at least one parent diagnosed with bipolar I disorder, who did not have any psychiatric disorder according to the K-SADS-PL-DSM-5-T administered by a clinician, and who consented to participate voluntarily along with their parents.

Exclusion Criteria for High-Risk Group:

1. Inadequate cognitive functions or intellectual levels to comprehend the tests.
2. Presence of other medical inflammatory disorders or psychiatric disorders.
3. History of alcohol or substance use.
4. Use of any medications that could affect cognitive functions and TRP metabolite levels.

The control group consisted of children aged 8-17 who did not have any psychiatric disorder according to the K-SADS-PL-DSM-5-T administered by a clinician and who consented to participate voluntarily along with their parents.

The study reached out to 242 patients diagnosed with bipolar disorder according to DSM-5 criteria through psychiatric interviews and follow-ups at the Psychiatry Department of Selçuk University Faculty of Medicine. Fifty patients were excluded because they did not have bipolar I disorder, and 108 were excluded because they did not have children aged 8-17. The remaining 84 parents, with a total of 94 children, were evaluated for participation. However, 17 children were excluded due to missing contact information, 16 parents declined participation, 13 children had other medical conditions, and 11 children were diagnosed with a psychiatric disorder according to the K-SADS-PL-DSM-5-T. Ultimately, the study group consisted of 37 children.

### Sociodemographic data form

The Sociodemographic Data Form is used to collect information about the age, educational status, ages of parents and family socioeconomic status (SES) of the children and adolescents participating in the study.

### Schedule for affective disorders and schizophrenia for school-age children-present and lifetime version

This semi-structured interview schedule is designed to assess past and current psychopathologies based on DSM-5 diagnostic criteria. The Turkish adaptation, which incorporates both dimensional and categorical diagnostic assessments aligned with DSM-5, was validated and deemed reliable in 2018. The interview schedule is systematically structured into three primary sections. The first section involves an unstructured interview conducted with the child and their family, aimed at gathering sociodemographic information, presenting complaints, developmental history, and an assessment of the child’s overall functioning at school and home. The second section comprises screening questions designed to evaluate over 200 specific symptoms, covering both past experiences and recent occurrences (within the last two months). Finally, the third section integrates assessment results with clinician observations to confirm diagnoses based on DSM-5 criteria. The K-SADS-PL-DSM-5-T facilitates the systematic screening of a wide range of primary psychiatric disorders. These encompass mood disorders, psychotic disorders, anxiety disorders, obsessive-compulsive disorder, neurodevelopmental and behavioral disorders, elimination disorders, eating disorders, tic disorders, substance-related disorders, as well as trauma- and stressor-related conditions (24).

### Stroop Test TBAG (TUBITAK Basic Sciences Research Group) form

The Turkish validity and reliability study for the Stroop Test TBAG form was conducted in 1999 (25). This test, used in the study, is a combination of the original Stroop Test and the Victoria Form. The Stroop Test TBAG Form is considered the gold standard for attention measurement. The Stroop Test is a neuropsychological assessment tool primarily designed to evaluate concentration and selective attention, while also providing insights into frontal region activity. Among neuropsychological tests, the Stroop Test is considered one of the most effective for assessing the ability to resist interfering stimuli, suppress inappropriate responses, shift targets in accordance with changing demands, and process information rapidly.The Stroop Test TBAG Form comprises five sections, each evaluated based on three parameters: duration, number of corrections, and number of errors. Duration reflects reaction time and the speed of information processing, corrections indicate impulsivity, and errors signify inattention. Prolonged durations and an increased number of errors and corrections are indicative of cognitive impairments, particularly in selective attention and information processing speed (26).

### Serial digit learning test

Developed by Zangwill, this test is used to evaluate short-term memory and learning ability. The Turkish validity and reliability studies for the SDLT were conducted in 1996 (27). The brain regions implicated in this test correspond to the mesial temporal lobe, frontal lobe, and hippocampus. SDLT has two variants: the 8-item and 9-item forms. During the test, the practitioner presents a sequence of numbers, and the subject is required to repeat the sequence in the same order. The test is concluded either when the subject successfully repeats the sequence twice consecutively or when all 12 attempts are exhausted. Performing this task, which involves learning a specific number and its spatial location, necessitates the engagement of various cognitive processes, including attention, information processing, memory, organization, and association (28). In this study, the 8-item form of the SDLT was utilized, emphasizing its application in evaluating these cognitive domains.

### Cancellation test

The CT consists of four subtests, which include both regular and irregular distributions of letters and shapes. These subtests are referred to as Regular Letters, Irregular Letters, Regular Shapes, and Irregular Shapes. Each subtest contains 60 target stimuli embedded within a total of 300 stimuli. In this test, participants are tasked with identifying the target stimuli and marking them by circling. Separate scores are calculated for each subtest: number of correctly marked targets (CT1), number of missed targets (CT2), number of incorrectly marked letters/shapes (CT3), total number of errors (CT4), and scanning time (CT5). The total error score is derived from the sum of missed target scores and incorrectly marked letter/shape scores. Developed by Weintraub and Mesulam in 1985, the Turkish version of the CT is one of seven separate neuropsychological tests used in the Cognitive Potentials’ Neuropsychological Test (BILNOT) Battery in Turkey (29). Although the test primarily evaluates selective attention and attention maintenance skills, it also enables the assessment of executive functions, including visual scanning, reaction speed, visual-motor coordination, retention of targets in memory, learning, strategy use, sequencing of planned responses, and maintaining behavioral set-up (30).

### Collection of biochemical samples and LC-MS/MS analysis

Blood samples were collected in a laboratory environment into gel vacutainer biochemistry tubes after at least 8 hours of fasting, in the morning. Blood samples from both the high-risk and control groups were centrifuged at 3000 rpm for 10 minutes. Plasma samples were collected into Eppendorf tubes and stored at -80°C until further processing. For deproteinization, 100 microliters of serum sample was mixed with 100 microliters of trichloroacetic acid containing an internal standard. The mixture was vortexed at 14,000 rpm for 5 minutes. Subsequently, 50 microliters of the supernatant was taken and used for analysis. The analyses were performed using the liquid chromatography-mass spectrometry (LC-MS/MS) equipment available within our hospital. Chromatographic separation was performed on a reverse-phase C18 column under gradient elution. The mobile phases consisted of 0.1% formic acid in water and 0.1% formic acid in acetonitrile. Mass spectrometric detection was conducted in positive ion mode using Multiple Reaction Monitoring. Analyte concentrations were calculated based on calibration curves. The method was validated for linearity, precision, accuracy, and sensitivity according to international standards.

### Statistical analysis

All data were evaluated using the SPSS-22 statistical program. Number, percentage, mean, and standard deviation were used in the evaluation of the data. The chi-square test was applied to two groups for categorical data. For numerical data, skewness and kurtosis values between -2 and +2 were used to comply with the normal distribution. When comparing the plasma tryptophan metabolites levels and neurocognitive properties of the high-risk and control groups, the student’s t-test was used for parameters with normal distribution, and the Mann–Whitney U test was applied for those with non-normal distribution. For all analyses, a significance value was accepted of *p*<0.05 at the 95% confidence interval. In addition, while examining the correlation and association between the cognitive characteristics of the high-risk group and plasma tryptophan metabolites, the data that fit the normal distribution were evaluated using Pearson’s correlation test, while the data that did not fit the normal distribution were evaluated with Spearman’s correlation analysis. Multivariate analyzes were planned to avoid type II errors due to multiple variables.

Socioeconomic status, BMI, age and sex were taken as covariates and blood levels of the groups were compared using MANCOVA. In addition, significant results from the cognitive tests were incorporated into the MANCOVA analysis. The Stroop 2nd Section Duration scores underwent square root transformation to meet the assumptions of parametric testing. However, CT2-FT scores were excluded from the MANCOVA analysis as they could not be normalized through any transformation. After observing a significant difference between the two groups with the MANCOVA test, one-way analysis of covariance (ANCOVA) was performed on the outcome variables separately. Effect sizes were estimated using Cohen’s d for parametric and non-parametric comparisons. Cohen’s d effect sizes were considered as ≥0.8 large, 0.5-0.7 intermediate, 0.2-0.4 small and <0.2 no effect (31).

## Results

The study included a total of 73 children and adolescents, comprising 37 unaffected high-risk group (17 boys, 20 girls) with a parent diagnosed with bipolar I disorder, and 36 healthy controls (23 boys, 13 girls). When the high-risk and control groups were compared in terms of age (t=-1.087, p=0.281), body mass index (t=-0.520, p=0.605), sex (x^2^ = 2,372, p=0.124) and SES (x^2^ = 3,154, p=0,207) no statistically significant differences were found between the two groups (see **Table 1**).

**Table 1:** Sociodemographic data of the high-risk and control groups.

	High-Risk Group n:32	Control Group n:31	t	P
**Age (Years)**	13,24 ± 2,39	12,61± 2,56	-1,087	0,281^a^
**BMI**	19,64± 3,15	19,21± 3,91	-0,520	0,605^a^
	N %	N %	x ^2^	p
**Sex**	Girl 20 60.6 Boy 17 42.5	13 39,4 23 57.5	2,372	0,124^b^
**Socioeconomic Status**	Low 24 64,9 Middle 13 35,1 High 0 0	18 50,0 16 44,4 25,6	3,154	0,207^b^

a Independent Sample T Test.

b Chi-Square Test, BMI, Body Mass Index. Socioeconomic status classification based on annual household income: Low (<$10,000/year), Middle ($10,000–$40,000/year), High (>$40,000/year). All individuals in the high-risk group had only one parent diagnosed with bipolar type I disorder.

When the two groups were compared in terms of Stroop test performance, it was found that only the completion time for the second part (z=-2.146, p=0.032) was statistically significantly higher in the high-risk group. Comparing the two groups in terms of SDLT performance, it was found that SDLT scores (t=2.05, p=0.045) had a statistically significant difference. The high-risk group had significantly lower SDLT scores compared to the controls. When the results of the CT were compared between the two groups, it was found that only the number of incorrectly marked targets in the regular shape (z=-2.298, p=0.022) was statistically significantly higher in the high-risk group (see **Table 2**).

**Table 2:** Neurocognitive test results of the high-risk and control groups.

	High-Risk Group N:37 Mean ± SD	Control Group N:36 Mean ± SD	t/z	p	d
**SD1**	11,62 ± 0,62	10,22 ± 0,34	-1,478	0,140^b^	0,27
**SE1**	0	0	0	1^b^	0
**SC1**	0,08 ± 0,04	0	-1,733	0,083^b^	0,28
**SD2**	12,03 ± 0,60	10,33 ± 0,39	-2,146	**0,032** ^b^	**0,33**
**SE2**	0,05 ± 0,05	0	-0,986	0,324^b^	0,14
**SC2**	0,02 ± 0,02	0,05 ± 0,03	-0,610	0,542^b^	0,08
**SD3**	15,54 ± 4,75	14,88 ± 3,60	-0,674	0,503^a^	0,01
**SE3**	0	0	0	1^b^	0
**SC3**	0,48 ± 0,69	0,41 ± 0,60	-0,460	0,647^a^	0.01
**SD4**	20,40 ± 1,47	20,67 ± 0,99	-0,944	0,345^b^	0,02
**SE4**	0	0,02 ± 0,02	-1,014	0,311^b^	0,11
**SC4**	0,70 ± 0,17	0,69 ± 0,11	-0,691	0,489^b^	0,005
**SD5**	28,45 ± 1,87	27,51 ± 1,11	-0,602	0,547^b^	0,06
**SE5**	0,43 ± 0,20	0,08 ± 0,06	-1,486	0,137^b^	0,23
**SC5**	1,70 ± 0,28	1,08 ± 0,14	-0,997	0,319^b^	0,27
**SDLT**	15,83 ± 6,82	18,55 ± 4,23	2,050	**0,045^a^**	**0,48**
**CT1-MaT**	57,18 ± 0,53	58,00 ± 0,36	-0,899	0,368^b^	0,17
**CT1-MiT**	2,81 ± 0,53	2,00 ± 0,36	-0,899	0,368^b^	0,17
**CT1-FT**	0	0	0	1	0
**CT1-TE**	2,81 ± 0,53	2,00 ± 0,36	-0,899	0,368 ^b^	0,17
**CT1-D**	116,45 ± 7,00	115,69 ± 0,43	-0,17	0,987^b^	0,01
**CT2-MaT**	58,32 ± 0,37	57,75 ± 0,44	-0,572	0,567^b^	0,14
**CT2-MiT**	1,72 ± 0,36	2,25 ± 0,44	-0,516	0,606^b^	0,12
**CT2-FT**	0,54 ± 0,17	0,08 ± 0,06	-2,298	**0,022** ^b^	**0,35**
**CT2-TE**	2,27 ± 0,43	2,44 ± 0,46	-0,051	0,960^b^	0,03
**CT2-D**	125,78 ± 8,74	116,72 ± 6,02	-0,248	0,804^b^	0,12
**CT3-MaT**	57,81 ± 2,15	58,22 ± 1,69	0,908	0,367^a^	0,02
**CT3-MiT**	2,16 ± 2,12	1,77 ± 1,69	-0,855	0,395^a^	0,01
**CT3-FT**	0,05 ± 0,03	0	-1,405	0,160^b^	0,23
**CT3-TE**	2,21 ± 2,13	1,72 ± 1,71	-1,090	0,279^a^	0,02
**CT3-D**	143,02 ± 11,02	139,75 ± 7,06	-0,679	0,497^b^	0,03
**CT4-MaT**	58,45 ± 1,67	57,97 ± 2,18	-1,067	0,290^a^	0,02
**CT4-MiT**	1,59 ± 1,69	2,02 ± 2,18	0,946	0,348^a^	0,02
**CT4-FT**	0,21 ± 0,08	0,19 ± 0,08	-0,257	0,797^b^	0,02
**CT4-TE**	1,81 ± 1,79	2,22 ± 2,21	0,880	0,382^a^	0,02
**CT4-D**	113,35 ± 10,13	108,77 ± 5,20	-0,536	0,573^b^	0,05

SD, Stroop duration; SE, Stroop error; SC, Stroop correction; SDLT, Serial Digit Learning Test; CT, Cancellation Test; MaT, marked target; MiT, missed target; FT, false target; TE, total error; D, duration. ^a^Student’s t-test, ^b^Mann–Whitney U test, Bold values indicate statistically significant results at p < 0.05., d: Cohen’s d.

In the comparison of arginine and its metabolites between the high-risk group and the control group, levels of ADMA, SDMA, and ornithine were significantly higher (t=-2.118, p=0.038; t=-2.858, p=0.006; t=-4.636, p<0.001, respectively) in the high-risk group compared to the controls. The citrulline level and the citrulline/arginine ratio, which indirectly reflect NOS activity, were found to be significantly lower in the high-risk group compared to the control group (t=8.54, p<0.001; z=-6.83, p<0.001) (see **Table 3**).

**Table 3:** Arginine and metabolite levels of the high-risk and control groups.

	High-Risk Group N:37 Mean ± SD	Control Group N:36 Mean ± SD	t/z	p	d
**Arginine**	32,34 ± 20,48	29,67 ± 18,77	-0,581	0,563^a^	0.135
**L-NMMA**	0,017 ± 0,008	0,015 ± 0,006	-1,069	0,289 ^a^	0.282
**ADMA**	0,229 ± 0,085	0,185 ± 0,091	-2,116	**0,038** ^a^	**0.502**
**SDMA**	0,293 ± 0,084	0,231 ± 0,101	-2,851	**0,006** ^a^	**0.667**
**Citrulline**	5,67 ± 2,97	29,03 ± 16,36	8,43	**<0.001** ^a^	**1.986**
**Homoarginine**	0,49 ± 0,22	0,51 ± 0,17	-0,932	0,351 ^b^	0.101
**Ornithine**	9,09 ± 3,56	5,29 ± 3,44	-4,638	**<0.001** ^a^	**1.085**
**Citrulline/Arginine** **(NOS Activity)**	0,828 ± 0,788	8,38 ± 9,22	-6,83	**<0.001^b^**	0.011

L-NMMA, n-monomethyl-l-argınıne acetate; ADMA, Asymmetric dimethylarginine; SDMA, Symmetric Dimethylarginine; NOS, Nitric Oxide Synthase ; a Student’s t-test, b Mann–Whitney U test, Bold values indicate statistically significant results at p < 0.05., d: Cohen’s d.

In the correlation analysis between Stroop test performance and arginine metabolites, a significant association was identified between the number of errors in the fifth part of the Stroop test and ADMA levels (rho = 0.369, p = 0.025). Furthermore, significant correlations were found between the durations of the first and second parts of the Stroop test and homoarginine levels (rho = 0.344, p = 0.037; rho = 0.408, p = 0.012, respectively). However, no significant relationships were observed between SDLT performance and arginine metabolites.

In the correlation analysis between CT data and arginine metabolites, several significant relationships were detected. L-NMMA levels were significantly correlated with the number of marked targets in the irregular letter section (r = -0.359, p = 0.029), the number of missed targets (r = 0.370, p = 0.024), and the total number of errors (r = 0.343, p = 0.038). Citrulline levels were associated with the number of marked targets in the irregular letter section (r = 0.358, p = 0.029), missed targets (r = -0.357, p = 0.03), total number of errors (r = -0.341, p = 0.039), and the total time for the irregular shape section (rho = 0.359, p = 0.029). Ornithine levels were significantly correlated with the number of incorrectly marked targets in the irregular letter section (rho = -0.336, p = 0.042), and NOS activity levels were associated with the number of missed targets in the irregular letter section (rho = -0.325, p = 0.05).

To minimize Type II errors resulting from multiple testing and to control for confounding factors such as sex, BMI, SES, and age, a MANCOVA test was performed. The MANCOVA test indicated a significant difference between groups (Pillai’s trace *V* = 0.773, *F* (9,59) = 22.272, *p* < 0,001, ηp² = 0.773). After observing a significant difference between the two groups with the MANCOVA test, one-way analysis of covariance (ANCOVA) was performed on the outcome variables separately. The same variables were taken as covariates in the ANCOVA analysis to determine which variables caused the differences between the groups. After controlling for SES, sex, age, and BMI, the comparison of SDLT, SD2, and the levels of arginine and its metabolites is presented in **Table 4**.

**Table 4:** Comparison of SDLT, Stroop test, Arginine and Metabolite levels in both groups according to ANCOVA.

Total samples ANCOVA^a^	High-Risk Group (n: 37)		Control Group (n: 36)		F (1.67)	P	η_p_^2^
	Mean	SD	Mean	SD			
**Arginine**	32,34	20,48	29,67	18,77	0,051	0,823	0,214
**L-NMMA**	0,01	0,00	0,01	0,00	0,797	0,375	0,035
**ADMA**	0,22	0,08	0,18	0,09	2,531	0,116	0,121
**SDMA**	0,29	0,08	0,23	0,10	7,682	**0,007**	0,214
**Citrulline**	5,67	2,97	29,03	16,36	62,531	**<0,001**	0,579
**Homoarginine**	0,49	0,22	0,51	0,17	0,300	0,586	0,081
**Ornithine**	9,09	3,56	5,29	3,44	18,097	**<0,001**	0,240
**Citrulline/Arginine (NOS Activity)**	0,24	0,18	1,84	1,90	19,400	**<0,001**	0,441
**SDLT**	15,83	6,82	18,55	4,23	4,568	**0,036**	0,079
**SQRT-SD2**	3,43	0,50	3,19	0,36	10,558	**0,002**	0,411

L-NMMA, n-monomethyl-l-argınıne acetate; ADMA, Asymmetric dimethylarginine; SDMA, Symmetric Dimethylarginine; NOS, Nitric Oxide Synthase. SQRT, square root; SDLT, Serial Digit Learning; Test; SD, Stroop duration;

a Covariates: Socioeconomic status; BMI, age and sex, Bold values indicate statistically significant results at p < 0.05..

## Discussion

The objective of this study was to explore the alterations in arginine and its metabolites in unaffected offspring of individuals diagnosed with BD compared to a control group. Additionally, we aimed to evaluate cognitive functions in these children and examine the potential relationships between these cognitive functions and the levels of arginine metabolites. Specifically, serum concentrations of arginine, L-NMMA, ADMA, SDMA, citrulline, homoarginine, ornithine, and NOS activity were measured in the high-risk offspring of BD patients and compared to those of the control group. Our findings indicated that the high-risk group exhibited significantly elevated levels of ADMA, SDMA, and ornithine, while citrulline levels and the citrulline/arginine ratio—an indirect measure of NOS activity—were significantly reduced in comparison to the control group. Moreover, the high-risk group demonstrated notable impairments in executive functions, which were correlated with elevated levels of ADMA, L-NMMA, citrulline, homoarginine, ornithine, and decreased NOS activity. These results suggest that arginine and its metabolites may play a contributory role in the underlying pathophysiology of BD.

MANCOVA analysis demonstrated that when SES, BMI, age, and sex were controlled as covariates, the significance of the cognitive data and arginine metabolites obtained in our study was largely preserved. Specifically, statistical significance was maintained for all metabolites and cognitive tests, except for ADMA. These results underscore the homogeneity of our study group and the reliability of the analyses, independent of these factors.

Moreover, previous studies have reported associations between SES, sex, BMI, and age with cognitive performance and metabolic processes (32–36). However, our findings highlight that the independent effects of arginine metabolism and cognitive functions remain evident even when these covariates are controlled. This suggests that arginine metabolism and cognitive processes may play distinct roles, and a homogeneous study group enables more robust and reliable analyses. However, which limits the ability to generalize across diverse populations. Future studies should aim to include larger and more diverse cohorts to facilitate subgroup analyses across SES, age, sex, and other relevant factors. Such analyses would provide a deeper understanding of how these variables interact with arginine metabolism and cognitive outcomes, thereby offering broader insights into their roles in bipolar disorder risk and progression.

Recent investigations into the role of arginine and related metabolic pathways in BD have focused on their influence on neurotransmitter regulation, neurovascular functioning, and cognitive processes (37). Nitrosative stress, resulting from an imbalance between RNS and antioxidant defenses, is a key factor in the pathophysiology of BD. Elevated RNS levels, such as peroxynitrite, cause oxidative damage to lipids, proteins, and DNA, leading to neuronal dysfunction, neuroinflammation, and synaptic impairment, which exacerbate mood and cognitive deficits in BD (5). ADMA contributes to nitrosative stress by reducing NO bioavailability. Increased ADMA, as observed in our study, disrupts endothelial function, neurovascular coupling, synaptic plasticity, and neurotransmission, further impairing mood regulation and cognition. Additionally, reduced NO levels, a critical regulator of neurovascular homeostasis and neurotransmitter systems, exacerbate neuroinflammation and neuronal dysfunction (8). The observed reductions in citrulline levels and NOS activity in the high-risk group support the role of impaired NO signaling in BD. The combined effects of elevated nitrosative stress and diminished NO levels create a pathological cycle that underlies BD’s clinical and cognitive symptoms. Targeting these pathways may offer promising therapeutic strategies for BD prevention and treatment. Elevated serum arginine levels have been reported in some studies, highlighting potential dysregulation in the urea cycle and arginine metabolism in BD patients (38). In addition elevated ADMA levels have been documented not only in BD patients but also in individuals with other psychiatric disorders (5). In parallel, increased levels of SDMA, ADMA, and arginine have been observed in individuals with BD and schizophrenia (39). L-NMMA, another inhibitor of NO synthesis, may modulate neurocognitive function through its effects on NO pathways, and thus could play a significant role in BD (17). Citrulline, which enhances arginine and NO levels, may contribute to neurological homeostasis; however, reduced citrulline levels have been documented in BD patients (40). NOS activity, which can be estimated by the citrulline/arginine ratio, is often diminished in BD, particularly nNOS activity in the central nervous system (41). Consistent with the findings of our study, emerging evidence supports the hypothesis that arginine pathway metabolites, such as ADMA, SDMA, and citrulline, may play a critical role in mood regulation and the pathogenesis of bipolar disorder (42).

Cognitive deficits in individuals with a familial predisposition to BD, particularly in verbal learning, memory, processing speed, and executive functions, have been well-documented, suggesting hereditary influences on cognitive functioning (43). In the present study, the Stroop test revealed that the high-risk group took significantly longer to complete the second section compared to controls, a result that aligns with previous literature. A recent study found that the offspring of bipolar parents performed below controls in areas such as processing speed, verbal fluency, visual memory, and working memory, with the greatest impairments observed in verbal memory and executive function (44). Performance on the SDLT indicated that the high-risk group showed poorer short-term memory and learning, lending further support to the notion that executive function deficits, particularly in verbal memory, may serve as an endophenotype for BD (45). The CT also indicated that the high-risk group made significantly more errors in target detection, further highlighting the executive dysfunction present in BD offspring.

A key hypothesis of this study was the relationship between arginine metabolites and neurocognitive functions. Prior research has demonstrated that NOS activity is associated with spatial memory (46). Furthermore, plasma ADMA levels have been found to be significantly elevated in patients with schizophrenia, with even higher concentrations in those experiencing multiple episodes. Notably, ADMA levels were shown to negatively correlate with attention, working memory, and executive function in schizophrenia patients (47). In individuals with major depressive disorder, a significant relationship has been observed between cognitive functions, particularly sustained attention, and the levels of ADMA and NO (48). Our findings revealed significant correlations between ADMA, L-NMMA, homoarginine, citrulline, ornithine levels, and NOS activity with executive and cognitive functions, which are in line with the literature. These results underscore the potential influence of arginine and its metabolites on cognitive performance, especially in high-risk individuals.

The strengths of our study include the comprehensive analysis of arginine metabolite levels in unaffected offspring of BD patients, making this the first study to examine these metabolites in relation to cognitive function in this specific population. We ensured sample homogeneity by carefully screening both high-risk and control groups for comorbid illnesses. Additionally, fasting serum samples were collected after an 8-hour overnight fast, minimizing dietary effects on metabolite levels. Several limitations of the study should be acknowledged. These include the relatively small sample size, the cross-sectional study design, the absence of direct NO measurements, the indirect assessment of NOS activity, and the exclusive evaluation of arginine metabolites in peripheral blood without consideration of central levels. Future longitudinal studies with larger sample sizes, incorporating cerebrospinal fluid analysis and neuroimaging techniques, are warranted to validate and further expand upon these findings.

In conclusion, our findings indicate that alterations in SDMA, citrulline, ornithine, and NOS activity in the unaffected offspring of BD patients may contribute to BD pathophysiology and serve as peripheral biomarkers in this high-risk group. Further longitudinal studies are needed to better understand these metabolic changes and their potential role in biomarker-based prevention strategies. This study highlights the link between arginine metabolism and neurocognitive function, offering valuable insights into BD endophenotypes and the early identification of at-risk individuals.

## Data Availability

The raw data supporting the conclusions of this article will be made available by the authors, without undue reservation.
